# Anticipatory coarticulation in non-speeded arm movements can be motor-equivalent, carry-over coarticulation always is

**DOI:** 10.1007/s00221-018-5215-5

**Published:** 2018-02-28

**Authors:** Eva Hansen, Britta Grimme, Hendrik Reimann, Gregor Schöner

**Affiliations:** 1 Institut für Neuroinformatik, Ruhr University Bochum, Bochum, Germany; 2 Department of Kinesiology and Applied Physiology, University of Delaware, Newark, USA

**Keywords:** Motor control, Movement planning, 3D human arm movements, Coarticulation, joint angles, End-effector trajectories, Uncontrolled manifold, Motor equivalence

## Abstract

In a sequence of arm movements, any given segment could be influenced by its predecessors (carry-over coarticulation) and by its successor (anticipatory coarticulation). To study the interdependence of movement segments, we asked participants to move an object from an initial position to a first and then on to a second target location. The task involved ten joint angles controlling the three-dimensional spatial path of the object and hand. We applied the principle of the uncontrolled manifold (UCM) to analyze the difference between joint trajectories that either affect (non-motor equivalent) or do not affect (motor equivalent) the hand’s trajectory in space. We found evidence for anticipatory coarticulation that was distributed equally in the two directions in joint space. We also found strong carry-over coarticulation, which showed clear structure in joint space: More of the difference between joint configurations observed for different preceding movements lies in directions in joint space that leaves the hand’s path in space invariant than in orthogonal directions in joint space that varies the hand’s path in space. We argue that the findings are consistent with anticipatory coarticulation reflecting processes of movement planning that lie at the level of the hand’s trajectory in space. Carry-over coarticulation may reflect primarily processes of motor control that are governed by the principle of the UCM, according to which changes that do not affect the hand’s trajectory in space are not actively delimited. Two follow-up experiments zoomed in on anticipatory coarticulation. These experiments strengthened evidence for anticipatory coarticulation. Anticipatory coarticulation was motor-equivalent when visual information supported the steering of the object to its first target, but was not motor equivalent when that information was removed. The experiments showed that visual updating of the hand’s path in space when the object approaches the first target only affected the component of the joint difference vector orthogonal to the UCM, consistent with the UCM principle.

## Introduction

Almost all natural activity that engages the arm and hand consists of sequences of individual movements. For instance, when you set the table, you perform a sequence of reaching movements to grasp and place plates and cups on the table surface. In a somewhat idealized version of such a task, we may consider the individual movements to be demarcated by moments in time at which the velocity of the hand in space comes close to zero, typically with a single peak of velocity in between. Each such individual movement is preceded and followed by other individual movements, which form the context for the given movement.

The fluidity of natural actions invites the question of how sequences of movements are planned and how the individual movement segments are coordinated. Are more than one segment planned ahead of time? Do those plans take into account the movement context?

The classical approach to these questions is to look for coarticulation, the influence of the movement context on each individual movement segment. Anticipatory coarticulation is the dependence of a movement on its successor, carry-over coarticulation is the dependence of a movement on its predecessor. Coarticulation is primarily known from speech articulatory movements whose high rate promotes the temporal overlap of articulatory gestures. In speech production, coarticulation is the influence of one phoneme on the sound and/or the articulator configuration of neighboring phonemes (e.g., Fowler and Saltzman 1993; Fowler 2014; Guenther 2016). Coarticulation often takes the form of assimilation in which an articulatory gesture is performed in a manner closer to the articulator configuration of the neighboring phoneme. Because of the challenges it implies for speech perception and for the existence of invariant units of speech, coarticulation has been an important topic in speech production.

Finger spelling in sign language reveals coarticulation that closely resembles the patterns observed for speech production (Jerde et al 2003). Both assimilation and dissimilation are observed, with some joints moving closer, others away from the configurations they have in preceding or subsequent gestures. Piano playing (Engel et al 1997), and touch-typing (Soechting and Flanders 1992) show signatures of anticipatory coarticulation in very limited form. Beyond finger movements, coarticulation has been seen in limb movements only under specific conditions (for review, see Grimme et al 2011). Klein Breteler et al (2003) examined 3D drawing sequences and found anticipatory coarticulation in the elevation in space of the elbow, but not in the end-effector path. Carry-over coarticulation was reported, for example, in an experiment in which participants sequentially touched locations on a table with an object (van der Wel et al 2007). When an obstacle positioned between two locations had to be avoided, the elevation of the object’s path on the subsequent movement segments was increased.

We previously reported an experiment with a somewhat similar task (Hansen et al 2015). An object was lifted up from a starting position and set down at a first target location, then lifted and moved to a second target location. This was unrestrained movement of the arm that made free use of the ten degrees of freedom of the arm to control the position of the object in space. The redundancy of the arm movement in this task led us to ask whether carry-over coarticulation affected primarily combinations of joint angles that did not affect the object’s position in space (motor equivalent coarticulation) or if coarticulation affected joint angles across all degrees of freedom. We used the method of analysis of the “uncontrolled manifold” (UCM) (Scholz and Schöner 1999) to answer this question.

**Fig. 1:**
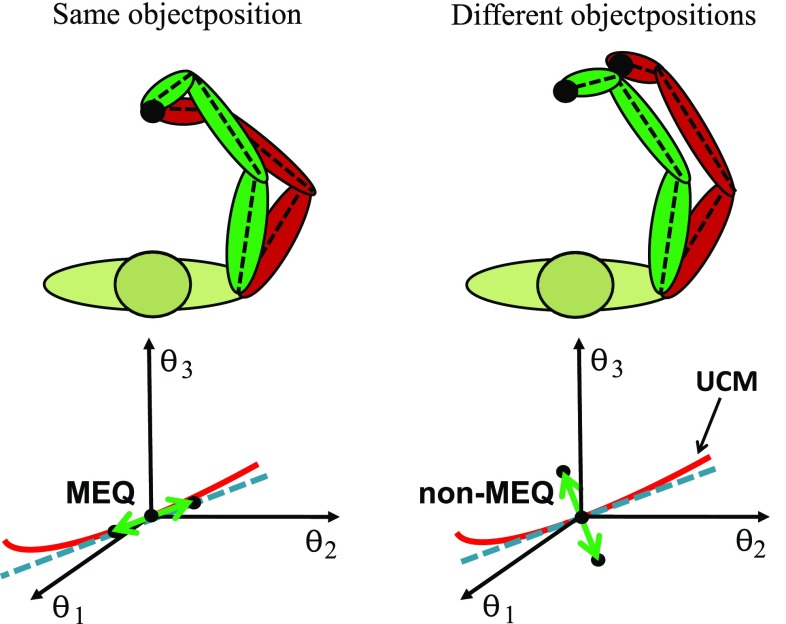
A planar three-joint arm may produce the two-dimensional position of the hand with different joint configurations (top left). This constraint defines a one-dimensional manifold, UCM, (solid line, bottom left) in joint space, that can be linearized (dashed line). Joint configurations that differ within the linearized UCM subspace are motor equivalent (MEQ). Joint configurations whose difference lies orthogonal to the linearized UCM subspace (shown on the right) lead to different hand positions and are not motor equivalent (non-MEQ)

The concepts behind this approach are illustrated in Fig. 1 for a three-joint planar arm moving the hand in two spatial dimensions. Different arm configurations can be used to realize the same hand position (illustrated on the left). All such configurations lie on a line, the uncontrolled manifold (the solid line in the bottom panel). Configurations that do not lie on this line differ in hand position (illustrated on the right). When movement context leads to different arm configurations at any given moment in time, we may ask, if their difference lies largely within the UCM. That would be the motor equivalent (MEQ) realization of the hand position. Alternatively, the arm configuration may also differ outside the UCM. That would be a non-motor equivalent (non-MEQ) realization of hand position. A formal analysis makes use of the linear approximation to the UCM (dashed line). The difference vector between joint configurations observed under different contexts is decomposed into a component within the linearized UCM subspace (left) and orthogonal to the linearized UCM subspace (right). Comparing the two components, we found in our earlier work (Hansen et al 2015) a sizeable difference primarily within the UCM subspace proving that carry-over coarticulation involves motor equivalent joint configurations. We argued that this form of carry-over coarticulation was consistent with prior reports about the dependence of joint configurations on the starting position of the hand (Cruse et al 1993) and largely reflect the control of multi-degree of freedom movements rather than its planning.

Anticipatory coarticulation was not observed. The task of Hansen et al (2015) did not promote anticipatory coarticulation, however, as the second movement target was visible throughout the trial so that there was no need for participants to recognize or prepare that second movement ahead of time. This raises the question, if “true” coarticulation does actually exist in such tasks, that do not involve rapid and highly learned movement sequences. True coarticulation would imply concurrent preparation of more than one movement segment and the resultant interdependence among subsequent movement segments. Movement plans must include the end-effector’s spatial position and path, which are critical, of course, to moving the hand. Therefore, true coarticulation is predicted to have a significant component orthogonal to the UCM, that affects the end-effector’s position in space rather than to lie predominantly in the UCM.

The experiments reported in this paper were designed to allow both for anticipatory and carry-over coarticulation in a redundant effector system. By comparing the structure of coarticulation in joint space we aim to uncover a difference between coarticulation that results primarily from motor control (leading to motor equivalent movement paths) and coarticulation that results primarily from movement planning (leading to differences in joint configuration in both subspaces). Anticipatory coarticulation is expected to result from movement planning and thus predicted to contain both components of joint space. Carry-over coarticulation may come from either level. A stronger component in the motor equivalent UCM subspace would indicate that carry-over coarticulation results in large part from the level of motor control.

The experimental paradigm we use is similar to the earlier one (Hansen et al 2015) and involved different sequences of two movements segments that transported an object from an initial location to a first and then to a second location on a table surface. To promote anticipatory coarticulation, we presented to the participants the two sequential movement targets for 3 s, but extinguished that information before the start of the first movement segment. Participants were therefore required to attend to and memorize both elements of the movement sequence. Unlike most other studies of coarticulation (Engel et al 1997; Klein Breteler et al 2003), we did not speed the movements, allowing for comfortable speed for naturalistic movements. The task thus provided an opportunity to study anticipatory coarticulation in segments of limb movements whose speed and overlap does not necessarily require it.

## Methods

The experimental methods are very similar to those in Hansen et al (2015), in particular, as concerns data collection and processing, joint angle computation, the analysis of variance using the uncontrolled manifold, motor equivalence, and the statistical analysis of the data.

### Subjects

Ten participants (5 male, 5 female) participated in the main experiment (no. 1), with ages 22–35 years (mean $\,=27$, SD $\,=\pm {4.53}$). In the two supplementary experiments (nos. 2 and 3) we had another ten participants each (5 male, 5 female, mean age$\,=26$, $SD\,=\pm 4.46$ for exp. 2; 2 female, 8 male, mean age$\,=29$, $SD\,=\pm 3.05$ for exp. 3). Two persons participated in both experiment 1 and 3. Except for one participant of experiment 3 (an author), all participants were unaware of the purpose of the study. All subjects were healthy and right-handed by self-report.

### Experimental setup

In all experiments participants performed a two segment movement task, in which they lifted and moved a cylindrical object (diameter = 6 cm, height = 15 cm, made out of Styrofoam with a small wooden middle section) from an initial location to a first, and then a second target location marked as circular targets on a horizontally arranged monitor.

**Fig. 2:**
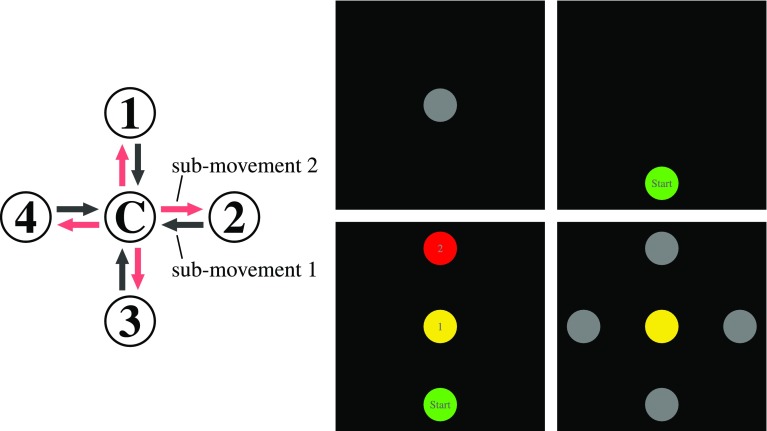
Left: Participants move a cylindrical object from one of the four positions 1–4 (exp. 1) or from position 3 (exp. 2 and 3) to the center C (first sub-movement) and then on to one of the four positions 1–4 (sub-movement 2). Right: Time course of the screens on the horizontal computer display on which participants lifted and touched down the object: Top left: Trials start with the center position in grey, where the object is first set down. Top right: The starting position is signaled in green. Bottom left: The cue to the movement sequence indicates the starting position in green, the first target in yellow, the second target in red. The cue lasted 3 seconds. The center was always the first target. Bottom right: With the go-signal, the four possible second target locations were shown in grey, while the center location remained yellow (exp. 1/2). In experiment 3, the screen turned black instead

Four locations (1–4) arranged around a central location (C) (illustrated in Fig. 2 on the left) were possible starting or target positions. The outer locations had a distance to the central location of 15 cm (measured from the midpoints of the circular disks).

In each trial, the participants first moved the object from one of the starting locations 1–4 to the center C (first sub-movement), touched the object down on that center location, and then moved the object to one of the target locations 1–4 (second sub-movement). For the first experiment any of the four outer circles were possible starting and final locations. This led to 16 conditions, each repeated twelve times. For the second and third experiment any of the four outer circles were possible final location but only location 3 was a possible starting location. Here four conditions with 50 repetitions were performed.

Participants sat on a chair in front of a monitor-table that was mounted horizontally. Their body midline was aligned with the center target position. They were secured to the chair with a harness that allowed normal scapular motion but prevented participants from moving their torso. For each participant, the experiment took about 1.5 h.

The computer screen guided participants through the task as illustrated on the right of Fig. 2. To start a trial, the center target lighted up in grey inviting participants to position the object at the center target. They were instructed to keep their grasp of the object invariant from this moment on. Next, one of the four starting locations appeared in green on the screen. Participants moved the object to that starting position and set it down there. The next screen of the display then informed participants about the sequence to perform. This screen was shown for three sec and indicated the first target, which always was the center, in yellow, and the second target in red. The next change of the screen was presented with an auditory “go” signal, authorizing participants to start moving, first to the center, touching the object down, then to the cued movement target. In experiments 1 and 2, the central target remained on in yellow, and all outer locations lit up in gray. In the third experiment, the display simply turned black. Participants were instructed to produce the cued movement sequence to the now invisible targets.

### Data collection and processing

Movements were recorded with the Visualeyez (Phoenix Inc.) motion capture system VZ 4000 using three tracker units containing three cameras each. Four rigid bodies (thermoplast molds) were placed and fixed with adhesive tape onto the four segments of the right arm as follows: (1) slightly to the left of the acromion process to acquire clavicle/scapula motion; (2) on the lateral part of the upper arm near the elbow joint; (3) on the dorsal side of the forearm near the wrist joint; and (4) n the dorsal side of the hand. Three active markers (wired infrared light emitting-diodes—IREDs) were attached to each rigid body, enabling the estimate of its 3D-position and orientation. A single marker was attached slightly above the sternum notch, and served as a reference position for joint angle computation. The object itself was tracked using a wireless IRED marker attached to its top.

The 3D trajectories of all markers were registered at a sampling rate of 101 Hz. All time series were filtered with a second-order zero-phase forward and reverse Butterworth filter with a cutoff frequency at 5.5 Hz. After filtering, the onset and termination times of each sub-movement were estimated from the trajectory of the object-IRED based on tangential velocity, acceleration, and distance to the starting position. Sub-movement that were recorded incompletely due to marker occlusion or other errors were dropped (9.16 %). Sub-movement trajectories were time normalized so that time is measured in percent of the mean movement time.

### Joint angle estimation

We established a kinematic model of the upper extremity using the product of exponentials formalism (Murray et al 1994). This model had three degrees of freedom (DoF) at the sternoclavicular joint, three DoFs at the shoulder joint, one DoF at the elbow, one DoF at the radioulnar joint and two DoFs at the wrist joint, for a total of 10 DoF. The marker at the sternum was used as a reference for all calculations.

The joint centers of rotation (CoR) were estimated from calibration movements that consisted of shoulder protraction/retraction and elevation/depression (sternoclavicular joint), shoulder flexion/extension and abduction/adduction (shoulder joint), elbow flexion/extension (elbow joint), pronation/supination of the lower arm (radioulnar joint) and wrist flexion/extension and abduction/adduction (wrist joint), with five repetitions each. The centers of rotation of the shoulder and wrist joints were estimated using the symmetrical CoR estimation algorithm (Ehrig et al 2006, SCoRE,). For the CoR of the sternoclavicular joint, a simple least-squares sphere fit was used because it reduced the estimation error compared to the SCoRE method. Similarly, the axis of elbow flexion-extension was estimated by the normal of a plane that optimally fit the trajectories of the lower arm markers during the calibration movement in the least squares sense.

To obtain the joint angles from the positions of the markers, we applied a global optimization method that minimized the summed squares of the distances between the measured marker positions and the positions reconstructed from the joint angles and the kinematic model (Lu and O’Connor 1999). The corresponding non-linear optimization problem was solved in Matlab using a generic gradient descent algorithm. The initial value was determined by calculating the joint angles from rotation matrices using standard techniques (Söderkvist and Wedin 1993). To characterize the accuracy of this estimation, we compared the marker position of the hand predicted from the estimated ten joint angles to the measured marker positions of the hand. Their difference was very small for all three experiments: ${2.34}\,\hbox {mm}$ (SD $\pm {2.42}\,\hbox {mm}$) for experiment 1; ${2.15}\,\hbox {mm}$ (SD $\pm {1.58}\,\hbox {mm}$) for experiment 2; ${2.10}\,\hbox {mm}$ (SD $\pm {1.85}\,\hbox {mm}$) for experiment 3. The marker on the object is not part of the formal analysis and is used for illustration only.

### Uncontrolled manifold and motor equivalence

We used the concept of the uncontrolled manifold (UCM) to analyze the structure of variance in joint space (Scholz and Schöner 1999). The method is reviewed in Schöner and Scholz (2007), Scholz and Schöner (2014) and illustrated in Fig. 1. It is based on decomposing joint configurations into the subspace within which a task variable is invariant (null-space, linear approximation to the UCM) and its orthogonal complement. Variance across repetitions at any point during the movement is then projected into either subspace and the two components of variance are compared. For the UCM analysis of variance we used the 3D position of the object as a task variable (compare Tseng et al 2003). In another test, we combined the 3D position with the orientation of the object in 2D (discarding rotation around the vertical long axis of the object) (compare Gera et al 2010).

To analyze coarticulation in joint space, we used the concept of the motor equivalence, also based on the uncontrolled manifold (Hansen et al 2015). The idea is to compare the joint trajectories generated in a sub-movement when the previous (carry-over coarticulation) or the subsequent (anticipatory coarticulation) sub-movement differs. This comparison across two different movement contexts consists of computing the difference vectors in joint-space and projecting them to either of the two subspaces, UCM and its orthogonal complement. Specifically, for two different movement contexts, *C*1 and *C*2, we compute at any given movement time *t*, the difference,

1 $$\begin{aligned} \varDelta \theta (t) = \overline{\theta }^{(C1)}(t) - \overline{\theta }^{(C2)}(t) \in {\mathbb {R}}^{10} \end{aligned}$$

of the joint configurations, $\overline{\theta }^{(C1)}(t)$, and $\overline{\theta }^{(C2)}(t)$ (means across trials). This difference vector is projected onto the null-space parallel to the UCM,

2 $$\begin{aligned} \varDelta \theta _\Vert (t)= E_\Vert (t) E_\Vert ^T (t) \varDelta \theta (t) \end{aligned}$$

and its orthogonal complement

3 $$\begin{aligned} \varDelta \theta _\perp (t) = E_\perp (t) E_\perp ^T (t) \varDelta \theta (t), \end{aligned}$$

where $E_\Vert (t) \in {\mathbb {R}}^{10 \times 7}$, and $E_\perp (t) \in {\mathbb {R}}^{10 \times 3}$ are spanned by the associated orthonormal vectors. These were determined by singular value decomposition of the Jacobian matrix, $J(t)= \partial \mathbf p / \partial \theta$, of object position $\mathbf p \in {\mathbb {R}}^3$. The Jacobian was evaluated at the average joint configuration across the two conditions. Given how little the Jacobian varies between the two conditions, this approximation has no discernible effect. The length of the difference vectors within each subspace was normalized by the dimensionality of either subspace, $d_\Vert =\Vert \varDelta \theta _\Vert \Vert /7$ and $d_\perp = \Vert \varDelta \theta _\perp \Vert /3$.

### Statistical analysis

The significance of the difference between the two components of variance (UCM and its orthogonal component ORT) was determined using a repeated measures analysis of variance (ANOVA) as implemented in SPSS. This was based on the means across trials for each participant and condition.

Statistical analysis of motor equivalence across context is hampered by the fact that the lengths, $d_\Vert$ and $d_\perp$, of the projections of the joint difference vectors onto the two subspaces are positive measures. They are thus sensitive not only to the mean of the underlying distribution, but also to its width. This leads to a contamination of the motor equivalence effect by the UCM effect of variance, as discussed in some detail in Hansen et al (2015). To avoid this problem, we analyze statistically the difference vectors themselves using multivariate analysis of variance (MANOVA) implemented in MATLAB. The MANOVAs detect differences between the projections of the vectors onto UCM (a 7-dimensional vector) and differences between projections of the vectors onto ORT (a 3-dimensional vector) across different context conditions. We report the outcome of these analysis in terms of the percentage of comparisons that led to significant outcomes, thus compressing the assessment of the statistical differences to a binary variable for each MANOVA. We report the outcome of these analyses in terms of the percentage of comparisons that led to significant outcomes, thus compressing the assessment of the statistical differences to a binary variable for each MANOVA.

Such an analysis is only meaningful, however, if the UCM and ORT spaces are similar across the compared movement contexts. We verified this by calculating the angle, $\gamma$, between the linearization of each UCM at different points in normalized time (1, 25, 50, 75 and 100%). That was small for all possible comparisons ($\overline{\gamma }= 0.031 \mathrm {rad}$; $SD=\pm 0.022 \mathrm {rad}$; maximum value$= 0.15 \mathrm {rad}$).

For both ANOVA and MANOVA, alpha was set at $P = 0.05$. The P values were adjusted for multiple comparisons using Bonferroni correction.

## Results

### Experiment 1

Experiment 1 probed both anticipatory and carry-over coarticulation and contained 16 different combinations of two sub-movements (Fig. 2). The first sub-movement starting from one of the four outer targets and going to the central target had a mean movement time of the end-effector of $0.55\hbox {s}$. Peak velocity was $0.59\,\hbox {ms}^{-1}$ ($SD=\pm \,0.069$). The second sub-movement went from the central target and to another of the four outer targets. Its mean movement time was $0.53 \hbox {s}$. Peak velocity was $0.56\,\hbox {ms}^{-1}$ ($SD=\pm \,0.057$). The touch-down between the two movement segments was very short in duration, too short to be reliably estimated.

#### Uncontrolled manifold analysis of variance

**Fig. 3:**
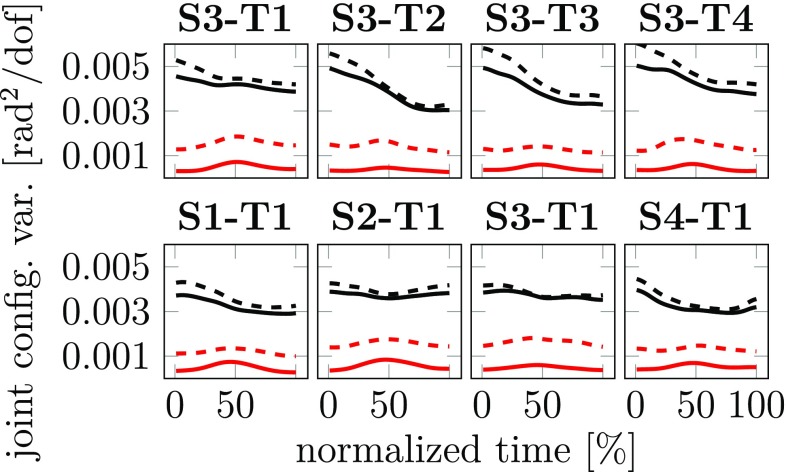
UCM structure of joint variance averaged across repetitions and participants for typical conditions. All first sub-movements that start from S3 are shown on top, all second sub-movements going to T1 are shown on bottom. For the task variable “3D position” of the object, the two components of joint configuration variance per DoF are shown as solid lines (UCM: black; ORT: red/grey line). For the task variable “3D position and 2D orientation”, the two components are shown as dashed lines

We analyzed the variance of joint configurations within (UCM) and orthogonal (ORT) to the uncontrolled manifold that reflects the task variable “3D position of the object”. Figure 3 illustrates these two components as a function of normalized time for a subset of conditions. UCM variance clearly exceeded ORT variance throughout the movement for both the first and the second sub-movement. The structure of variance was very similar for the other conditions.

To statistically analyze the UCM structure of variance, we picked 5 equidistance points in normalized movement time, (1, 25, 50, 75 and 100%). For the first and second sub-movement, separate repeated measures ANOVAs were performed with the three factors time, condition, and component (UCM vs. ORT). The UCM component was significantly larger than the ORT component in both the first ($$F_{1,9}=68.042,~p<0.001$$) and the second ($$F_{1,9}=45.419,~p<0.001)$$ sub-movement. For both sub-movements, an interaction between the time and component was detected. Therefore, follow-up analysis was performed at each of the five points in time for the two sub-movements. The UCM component was significantly larger than the ORT component at all points in time.

Figure 3 also illustrates two components of joint configuration variance with respect to the task variable “3D position and 2D orientation” of the moving object. The UCM effect is very similar to that of the 3D position hypothesis, with a slightly larger ORT component. This pattern was consistently observed across all 32 sub-movements. This illustrates that the UCM effect of position was not primarily caused by variation in the object’s orientation. Because keeping the object oriented vertically was not explicitly part of the task, we do not further report on this second hypothesis.

#### Coarticulation at the level of end-effector paths and trajectories

**Fig. 4:**
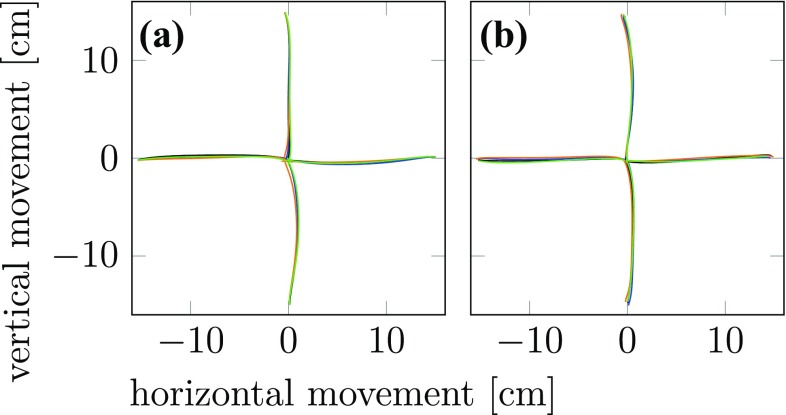
End-effector paths from Experiment 1 (means across all repetitions and participants) are projected onto the table-plane. **a** Movement paths for the first sub-movement going from the four starting targets to the center target are shown in different colors for the different conditions in which the second sub-movement goes to different final targets. **b** Movement paths of the second sub-movement going from the center target to the four outer targets are shown in different colors for the different conditions in which the first sub-movement comes from different starting targets

To visually inspect end-effector paths for signatures of coarticulation, we plotted the horizontal coordinates of the marker position on the object for two cases. Illustrating anticipatory coarticulation, Fig. 4a shows the mean two-dimensional object paths for the first sub-movement leading to different second targets. These paths are not distinguishable, so at first glance there is no evidence for anticipatory coarticulation at the level of the object path (but see below). Illustrating carry-over coarticulation, Fig. 4b shows the mean two-dimensional object paths for the second sub-movement that were preceded by different first sub-movements. Again, no differences are visible to the naked eye, suggesting that carry-over coarticulation is not detectable at the level of the object paths.

**Fig. 5:**
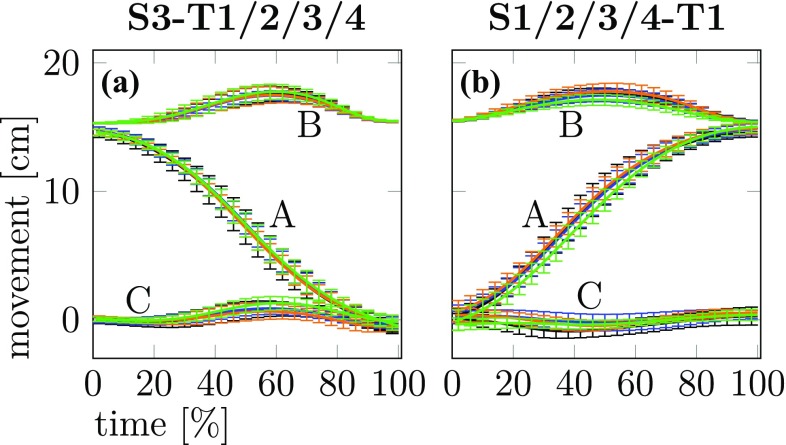
The three Cartesian components of the end-effector trajectories averaged across repetitions are shown as a function of normalized movement time for a typical participant together with error bars that represent the standard deviation across repetitions. A: Coordinate along an axis pointing from the initial to the final end-effector position B: Coordinate along an axis pointing up vertically. C: Coordinate along an axis that is orthogonal to A and B. **a** Trajectories for the first sub-movement with different colors for the four conditions, S3-T1/2/3/4. **b** Trajectories for the second sub-movement with different colors for the four conditions, S1/2/3/4-S1

The end-effector trajectories across time can also be examined for coarticulation effects. Figure 5 shows the three Cartesian components of the end-effector as a function of time together with error bars that mark the standard deviation at sampled points in time. These data from a typical participant and show movement from one starting target (S3 on the left) and to one final target (T1 on the right). The four different movement contexts for the first and for the second sub-movement are color coded. Clearly, differences between the end-effector trajectories with different movement context are small, lying within a standard deviation at all times. We observed the same pattern for the other possible comparisons and the other participants.

#### Coarticulation at the level of joint angle trajectories

**Fig. 6:**
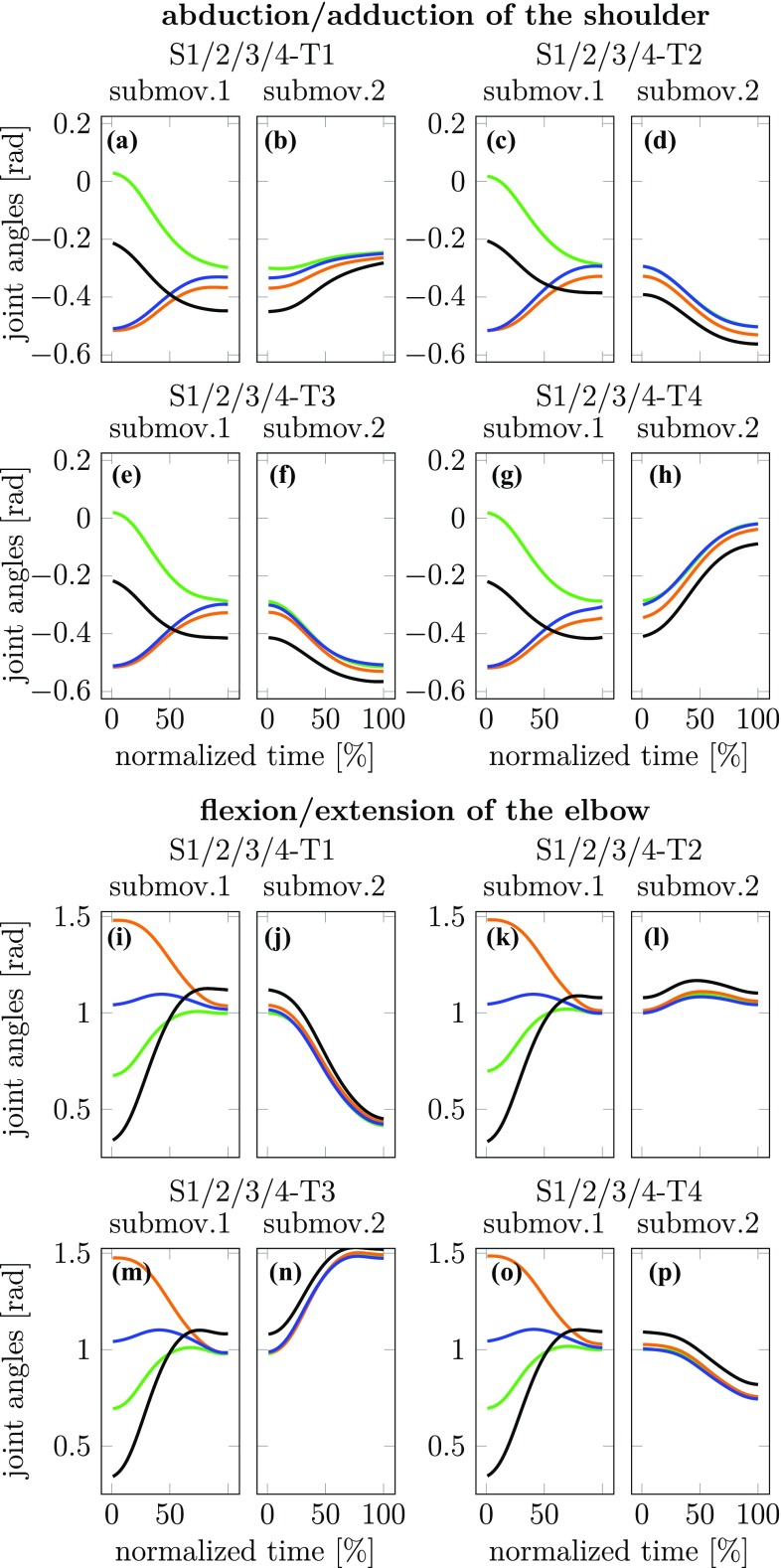
Joint angle trajectories (averaged over repetitions and participants) for the two joint angles abduction/adduction of the shoulder (**a**–**h**) and flexion/extension of the elbow (**i**–**p**) are displayed for experiment 1. For either joint, the pairs of plots of first and second sub-movements (e.g., **a** and **b**) shows the joint trajectories for movements going to the same final target from either of the four different starting targets (color coded). This same illustration is provided for each of the four final targets

**Fig. 7:**
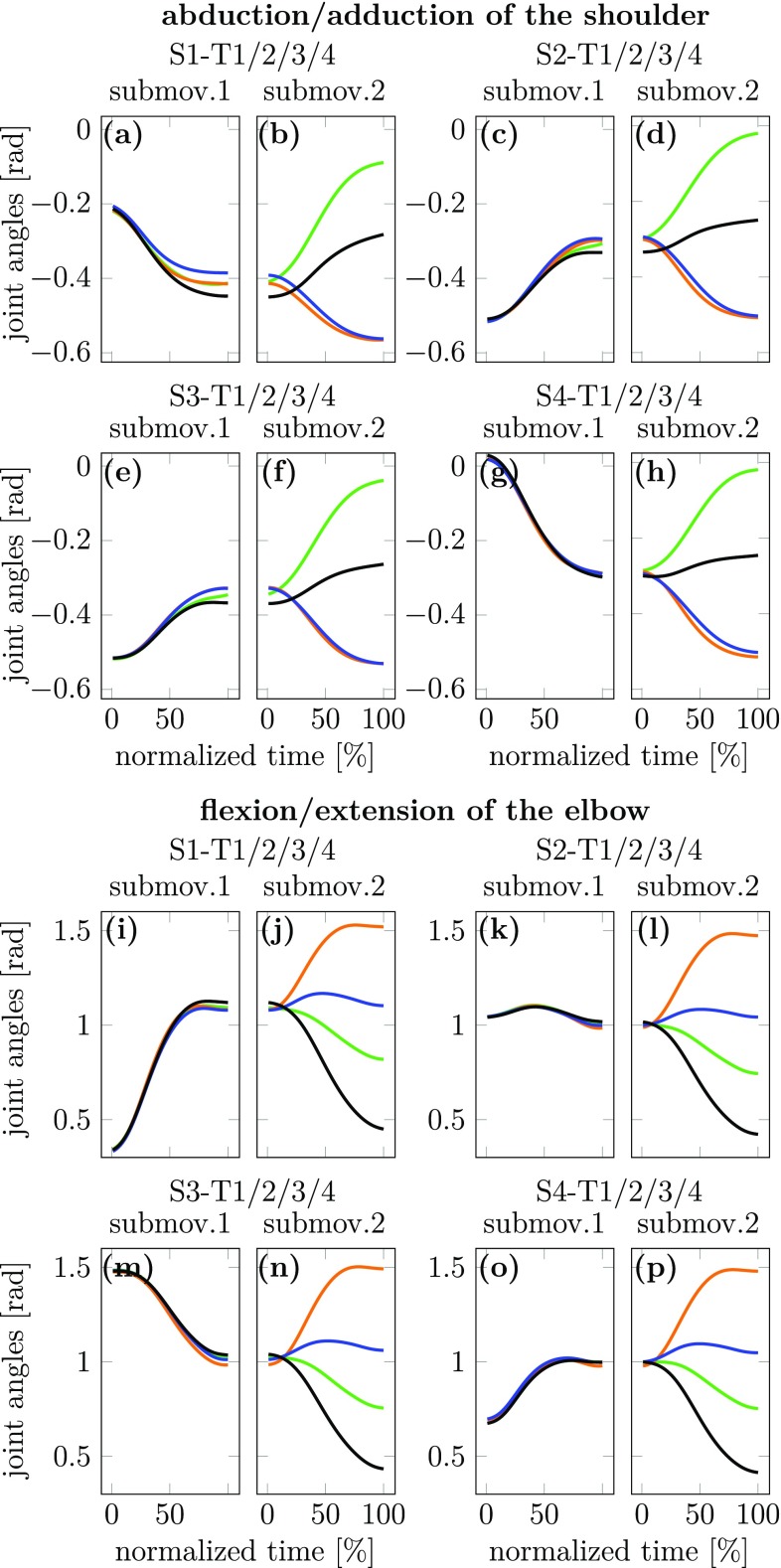
Joint angle trajectories (averaged over repetitions and participants) for the two joint angles abduction/adduction of the shoulder (**a**–**h**) and flexion/extension of the elbow (**i**–**p**) are displayed for experiment 1. For either joint, the pairs of plots of first and second sub-movements (e.g., **a** and **b**) shows the joint trajectories for movements from the same starting target going to each of the four different final targets (color coded). This same illustration is provided for each of the four starting targets

Joint angle trajectories may be revealing of coarticulation effects. We illustrate this in Figs. 6 and 7, in which we show the time courses of two out of ten joint angles (abduction/adduction of the shoulder and flexion/extension of the elbow). Carry-over coarticulation is visible from Fig. 6 that shows the movements to the four final targets, color-coding for the four different starting targets. The first sub-movement reflects these different starting targets. Note that the joint configurations do not fully converge at the end of the first sub-movement, so that the center target is reached with slightly different joint configurations. These differences largely persist during the second sub-movement, thus exhibiting carry-over coarticulation.

Anticipatory coarticulation is visible in Fig. 7 that shows movements from the four starting targets, color-coding for the four different final targets. The second sub-movement reflects these different final targets. The first sub-movement differs for the different final targets, more strongly so for the shoulder than for the elbow. This is suggestive of anticipatory coarticulation. Note, that the different joint configurations reached after the first sub-movement do not persist during the second sub-movement. In this sense, anticipatory coarticulation is both weaker and different from carry-over coarticulation.

Similar patterns can be found for the other eight joint angles.

#### Motor equivalence analysis to detect coarticulation

**Fig. 8:**
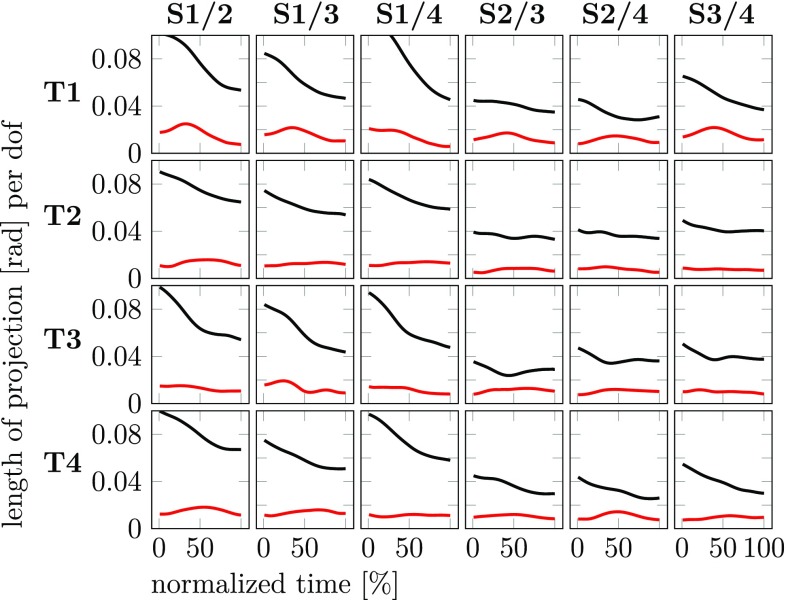
The lengths of the two components of difference joint vectors, MEQ, $d_\Vert$ (black) and non-MEQ, $d_\perp$ (red), across pairs of movement contexts are shown for the second sub-movement as functions of normalized movement time (experiment 1, averages over participants, to detect carry-over coarticulation). Each row shows comparisons with the same final target. The columns depict all possible comparisons across pairs of different starting targets

A more comprehensive analysis of coarticulation in joint space makes use of the comparision across different movement contexts using the concept of the uncontrolled manifold. To analyze carry-over coarticulation we compare the second sub-movement going to the same final target across different first sub-movements coming from different starting targets. For each final target, there are four starting targets leading to six pairwise comparisions. We assess coarticulation in terms of the two components of the difference joint vector across the pair of movement context: motor equivalent (MEQ) and non-motor equivalent (non-MEQ). The lengths of these two components are shown in Fig. 8 across time. There is consistently more MEQ difference than non-MEQ difference, suggesting that carry-over coarticulation lies largely in the UCM subspace. The MEQ component tends to decrease over the second sub-movement, reflecting a gradual decay of the carry-over effect.

**Fig. 9:**
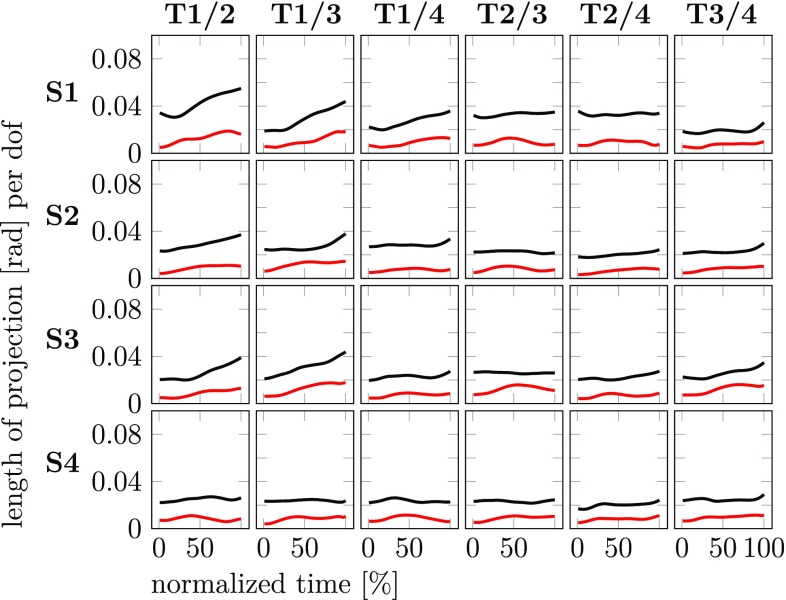
The lengths of the two components of difference joint vectors, MEQ, $d_\Vert$ (black) and non-MEQ, $d_\perp$ (red), across pairs of movement contexts are shown for the first sub-movement as functions of normalized movement time (experiment 1, averages over participants, to detect anticipatory coarticulation). Each row shows comparisons with the same starting target. The columns depict all possible comparisons across pairs of different final targets

Anticipatory coarticulation is detected by comparing pairwise the first sub-movement coming from the same starting target going to different final targets. For each starting target, all six such pairwise comparisions are shown in Fig. 9. The length of the MEQ component is consistently above the length of the non-MEQ component across time and conditions suggesting that anticipatory coarticulation also lies more within the UCM than orthogonal to it. Note, however, that the length of the MEQ component ranging from around 0.02–0.05 rad/DoF is overall smaller than the corresponding length for carry-over coarticulation that ranges from 0.02 to 0.1 rad/DoF. The length of the non-MEQ component in the two forms of coarticulation is similar: 0.003–0.02 rad/DoF for anticipatory and 0.005–0.03 rad/DoF for carry-over coarticulation. Thus, carry-over coarticulation is stronger than anticipatory coarticulation, and that increased strength comes primarily from contributions that lie within the UCM.

#### Statistical analysis of motor equivalence

To statistically analyze motor equivalence we need to take into account that the underlying difference, $\varDelta \theta$, between the two mean joint configurations is a multi-dimensional vector. When that vector is compressed into a one-dimensional length measure within either of the two sub-spaces, two positive numbers result. Probability distributions of positive numbers cannot be normal and symmetric, of course. Means thus interact with variances: the broader the distribution, the larger the mean. Because the variance of joint configurations is larger in the UCM subspace than in the ORT subspace, the distribution underlying the MEQ component is broader than the distribution of the ORT component. As a consequence, observing a larger mean length of the MEQ component than of the ORT component could be caused by the different widths of the underlying distributions. For a detailed discussion and analysis of this problem, see Hansen et al (2015).

The solution we developed in this earlier work was to directly assess the joint difference vectors projected onto the two subspaces using MANOVAs (multivariate analysis of variance). This avoids taking the length measure that produces positive numbers and induces skewed distributions. The dependent variables of each MANOVA are the seven dimensions that span the UCM component of the joint angle difference vector in one case, and the three-dimensions that span the ORT component of the joint angle difference vector in the other case. The directions defining the UCM and the ORT subspaces are different for different subjects, so an analysis across participants would be meaningless. We therefore performed separate MANOVAs for each participant, entering the joint angle difference vectors observed on each trial. Separate MANOVAs were performed at five points in normalized time (1, 25, 50, 75, and 100%), for the first and the second sub-movement, and for the two components, UCM and ORT. The factor in these MANOVAs was the movement context. For anticipatory coarticulation observed on the first sub-movement this was the final target position with four levels (T1–T4). For carry-over coarticulation observed on the second sub-movement this was the starting position with four levels (S1–S4). *P* values were adjusted using the Bonferroni correction. Alpha was set at $P = 0.05$.

**Fig. 10:**
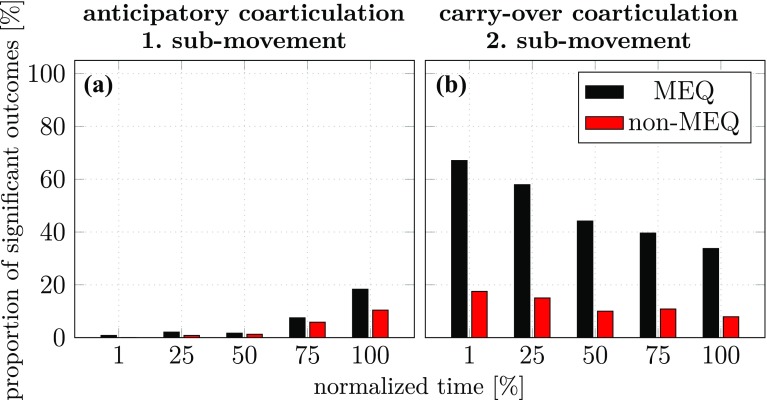
Percentage of MANOVAs that yield significant effects for anticipatory coarticulation (**a**) and carry-over coarticulation (**b**) at five points in time during the two sub-movements of experiment 1. Shown are outcomes of MANOVAs that used as dependent variable the joint configuration difference vector in the UCM space (MEQ, coarticulation at the level of joint angles) or the joint configuration difference vector in the ORT space (non-MEQ, coarticulation at the level of the end-effector)

In Fig. 10a and b we summarize the outcomes of these MANOVAs by reporting the percentage of MANOVAs that led to significant effects. The resulting proportions of significant outcomes cannot be interpreted as absolute values nor be compared across different experiments, especially where experiments differ in statistical power. Within an experiment, however, these proportions may tracked over time and can be used to compare different conditions. Part a of the figure shows the proportion of significant outcomes for anticipatory coarticulation across the five moments in time during the first sub-movement. The observed outcomes suggest that anticipatory coarticulation exists in both subspaces and increases over time during the first sub-movement. Part b of the figure shows the proportion of significant outcomes for carry-over coarticulation across the five moments in time during the second sub-movement. There is a much larger percentage of the proportion of significant outcomes, more strongly so for the MEQ component (carry-over coarticulation at the level of the joint angles) than for the non-MEQ component (carry-over coarticulation at the level of the end-effector). Both components decrease over time during the second sub-movement.

### Experiment 2

To further investigate anticipatory coarticulation, for which experiment 1 provided only weak evidence, we reduced the number of conditions from 16 to 4 (S3–T1/2/3/4) so that we could increase the number of repetitions from 12 to 50. All other parts of the experiment stayed the same. This design enabled the detection of anticipatory coarticulation only across six different pair-wise comparisons.

**Fig. 11:**
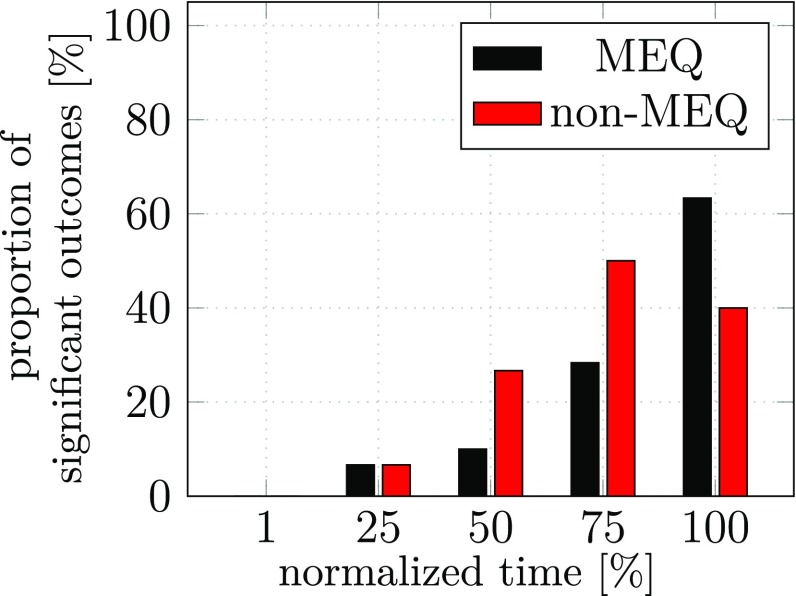
Percentage of MANOVAs that yield significant effects for anticipatory coarticulation at five points in time during the first sub-movement of experiment 2. Shown are outcomes of MANOVAs that used as dependent variable the joint configuration difference vector in the UCM space (MEQ, coarticulation at the level of joint angles) or the joint configuration difference vector in the ORT space (non-MEQ, coarticulation at the level of the end-effector)

The outcome of the same statistical analysis as in experiment 1 based on MANOVAs is shown in Fig. 11. The percentage of MANOVAs that led to significant effects is shown for either component across the five points in time of the first sub-movement. The larger number of trials in this experiment has increased the power of the MANOVA boosting the number of significant effects and providing stronger evidence for anticipatory coarticulation in both components, increasing over time. End-effector level anticipatory coarticulation (non-MEQ) is detected more frequently except for a drop of that form of coarticulation at the end of the first sub-movement.

### Experiment 3

The drop-off of evidence for anticipatory coarticulation at the level of the end-effector at the end of the first sub-movement found in experiment 2 was further investigated. This drop-off might have been caused by corrections of the hands path based on visual feedback as the object neared the center target. To test this explanation, we removed in experiment 3 all visual information about movement targets at the same time as the go-signal triggered movement initiation.

**Fig. 12:**
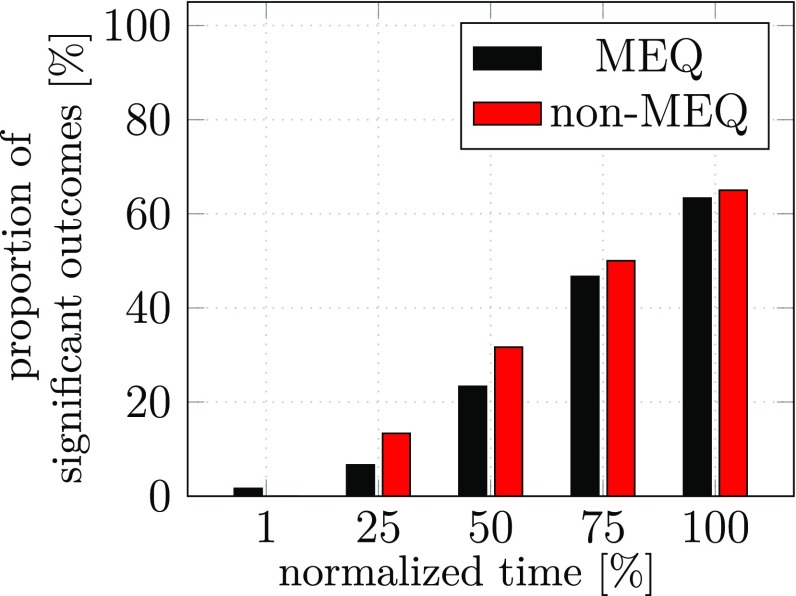
Percentage of MANOVAs that yield significant effects for anticipatory coarticulation at five points in time during the first sub-movement of experiment 3. Shown are outcomes of MANOVAs that used as dependent variable the joint configuration difference vector in the UCM space (MEQ, coarticulation at the level of joint angles) or the joint configuration difference vector in the ORT space (non-MEQ, coarticulation at the level of the end-effector)

The outcome of the statistical analysis using MANOVAs is shown in Fig. 12. Clear evidence for anticipatory coarticulation in both components is replicated, increasing over time during the first sub-movement. End-effector level coarticulation no longer drops at the final state of that sub-movement.

## Discussion

We set out to study coarticulation in naturalistic transport movements under conditions that made anticipatory coarticulation plausible and potentially observable at the same time as carry-over coarticulation. Participants moved a cylindrical object from an initial position to a center target and on to a final target. Visual information about the final target was removed at the beginning of the first sub-movement, forcing participants to memorize and potentially plan the second sub-movement ahead of time. This paradigm enabled replication of our earlier observation of carry-over coarticulation (Hansen et al 2015). In that earlier task, information about the final target was available throughout the movement sequence and also served as a go-signal, so that there was little time to pre-plan the second movement before movement initiation.

We successfully replicated carry-over coarticulation, but found new evidence for consistent, if small anticipatory coarticulation. The analysis made use of the redundant nature of the task that required moving the hand to targets in three dimensions engaging the 10 degrees of freedom of the arm. We used the concept of the uncontrolled manifold (UCM) to determine if coarticulation affected primarily the end-effector (non-motor equivalent, non-MEQ) or primarily the joint angle trajectories and not the end-effector (motor equivalent, MEQ). We replicated our earlier finding that carry-over coarticulation was more strongly observed in joint than in end-effector space. Anticipatory coarticulation, in contrast, was found equally in both subspaces. The time course of coarticulation reflected causation: anticipatory coarticulation increased as the second sub-movement is approached, carry-over coarticulation decreases with increasing distance in time from the first sub-movement.

### UCM effect of variance

Before investigating coarticulation, we examined the standard “UCM effect” of variance, that is, the hypothesis that more variance across trials is observed in directions in joint space that leave the hand’s position in 3D invariant than in directions in joint space that affect the hand’s 3D position (Scholz and Schöner 1999; Tseng et al 2002; van der Steen and Bongers 2011; Jacquier-Bret et al 2009; Mattos et al 2011). We replicated yet again a clear UCM effect (Fig. 3). The UCM structure of variance was observable from the start of each movement, probably due to the fact that we did not impose an invariant arm configuration before movement initiation. Variance within the UCM was largely invariant across time, suggesting that the number of DoF is not constrained by kinematic singularities or joint limits within the workspace used in the task. This makes the task suitable for an analysis of the structure of coarticulation in joint space. In some earlier studies (Tseng et al 2003; Scholz et al 2000), the ORT component of variance increases in the middle of a movement. Temporal misalignment across trials induces such variance which peaks when movement speed is maximal and affects primarily the ORT component. Figure 3 shows little modulation of the ORT component, underlining that temporal alignment was good.

### End-effector and joint angles trajectories

A direct search for signatures of both forms of coarticulation at the level of end-effector paths (Figs. 4 and 5) revealed no such effects. This shows that coarticulation may be hard to observe at this level and also provides a note of caution about failures to observe coarticulation in the literature.

Looking directly at joint angle trajectories does provide hints at carry-over coarticulation (Fig. 6). Two joint angle trajectories on the second sub-movement were found to differ when they came from different starting positions on the first sub-movement, a difference observable from the beginning of the second sub-movement and only decreasing slightly in time. Anticipatory coarticulation was difficult, however, to detect at this level (Fig. 7a–h).

Overall, joint angle trajectories are difficult to analyze as different joints behave differently and the trajectory shapes vary with movement target and joint. This provides a strong incentive for the UCM based method of analysis of coarticulation.

### UCM approach to coarticulation

Coarticulation is established by showing that which movement precedes (for carry-over coarticulation) or follows on (anticipatory coarticulation) a particular movement matters. In other words, differences between movement trajectories observed in different contexts are examined for significance. Are such differences, if they exist, primarily due to different trajectories of the end-effector, and thus perhaps related to the perceptual basis for movement or to its planning? Or are these differences primarily due to different joint angle trajectories and thus perhaps related to the control of the movement? We saw that there is no obvious answer to these questions from a direct examination of the end-effector or joint trajectories. Moreover, a direct comparison of the two types of differences does not make conceptual sense. Differences in end-effector trajectories are in three-dimensional Cartesian space and are measured in centimeters, differences in joint configuration live in ten-dimensional angular space and are measured in radians. The concept of the uncontrolled manifold (UCM) resolves this problem by embedding both differences in a shared embedding space, here joint-space, and employing a single metric within that space. The link between the two types of trajectories is provided by the kinematic model of the arm, which is given at any point in joint space by the arm’s Jacobian. The Jacobian translates, in a sense, changes in joint space into changes in Cartesian space. For the question of movement context, the Jacobian translates differences in joint angle vector across contexts into differences in end-effector space.

Specifically, the null-space of the Jacobian (UCM) and its orthogonal complement (ORT) are used to decompose difference vectors between joint configurations observed in different movement contexts. The UCM component indicates dependence on movement context that includes joint configurations that do not affect the end-effector. Differences that lie in the UCM are thus due to motor equivalent (MEQ) solutions to the movement problem when a particular movement is preceded or followed by different movements. The ORT component, in contrast, represents a difference that lies completely at the end-effector level. Joint configuration differences that do not affect the end-effector are minimized. Such differences are therefore reflective of non-motor equivalent (non-MEQ) solutions to the movement problem in different movement contexts.

The UCM concept has frequently been used to assess variance in joint trajectories. We have again confirmed that the UCM component of variance is larger than the ORT component at any moment in time along all sub-movements. To use the UCM concept to assess motor equivalence, we need to measure the size of the difference in the two subspaces. Using the length of the difference vector in each subspace, scaled to the dimensionality of each subspace, first seems an obvious choice and we illustrated these length measures in Figs. 8 and 9. On this basis, we observed strong carry-over coarticulation which lay primarily in the UCM, reflecting motor equivalent solutions (MEQ) to the second sub-movement, when different first sub-movements were compared. Anticipatory coarticulation was also observed, although much smaller in size.

Previously, we uncovered and solved a subtle problem in statistically assessing motor equivalence, that is particularly relevant for anticipatory coarticulation (Hansen et al 2015). Length is a positive measure, so that the underlying probability distribution is bounded below by zero. If the mean or the mode of that distribution lies close to zero compared to its width, then the mean of the length covaries with the variance of the underlying distribution: a broader distribution will have a higher mean. For anticipatory coarticulation, the mean length of the difference vector is small (roughly 0.01 rad for non-MEQ, 0.04 rad for MEQ). The standard deviation of the underlying distributions are larger (roughly 0.03 rad for ORT, 0.06 rad for UCM). So assessing anticipatory coarticulation is affected by this statistical problem. Specifically, the larger variance of the distribution in the UCM subspace will bias the estimate of the mean length in the UCM subspace toward larger value. Thus, the tendency for anticipatory coarticulation to be motor equivalent suggested by Fig. 9 could be spurious.

The solution to this problem developed in Hansen et al (2015) is to assess the difference across movement context within the two subspaces directly in terms of the difference joint vectors. This can be done using one-factor MANOVAs, into which the seven-dimensional (for MEQ) or three-dimensional (for non-MEQ) joint difference vectors enter. The factor is movement context. This analysis must be done separately for each participant, time, and sub-movement (compensating with Bonferroni correction). The percentage of these tests that detect significant differences is then used to measure coarticulation as a function of time and sub-movement as illustrated in Fig. 10a and b.

On this basis, we have replicated carry-over coarticulation (part b of the figure), with a strong signature of motor equivalence. When the arm makes different first sub-movements, the second sub-movements are motor equivalent, different more strongly in the joint trajectory than in the end-effector trajectory. This difference decreases over time, which is natural given that what causes the difference, the preceding sub-movement, lies increasingly further in the past.

We have also observed anticipatory coarticulation. This effect was small, however, not larger in this form of assessment than in our previous experiment (compare Figure 10 in Hansen et al 2015). In experiment 2 we further investigated this by increasing the number of trials from 12 to 50 per participant, focussing exclusively on the movement contexts that probe anticipatory coarticulation. That boosted the statistical power of our MANOVAs and yielded the results shown in Fig. 11. Evidence for anticipatory coarticulation is clear. The size of the effect increases as time approaches the second sub-movement that induces the effect. There is no systematic difference between MEQ and non-MEQ components. In fact, one curious signature of our earlier study (Figure 10 in Hansen et al 2015) is replicated: the non-MEQ component drops at the last moment in time of the first submovement. This might be due to visually guided corrections on the hand’s path to steer it to the first (center) target. Such a correction would reduce differences in the hand’s trajectory across movement contexts, and thus decrease the non-MEQ component of the difference joint vector.

To test this account, we removed visual information about all movement targets before the initiation of the movement in experiment 3, otherwise replicating the conditions of experiment 2. The results shown in Fig. 12 confirm the pattern of anticipatory coarticulation, but now remove the dip of the non-MEQ component at the last moment in time of the first sub-movement. This confirms our account and thus provides indirect evidence for such visually based steering of the hand to the center target. These results suggest that anticipatory coarticulation is motor-equivalent close to the end of the first segment when visual information helps steer the hand-held object to its target. When that information is removed, this compression of the non-MEQ coarticulation is no longer possible. Under those conditions, the two components of anticipatory coarticulation, MEQ and non-MEQ, are about equal in size.

### What does the observed pattern of coarticulation mean?

Anticipatory coarticulation is clearly reflective of motor planning. Anticipatory planning of movements has been observed in a number of paradigms (e.g., Herbort and Butz 2009). We have found that anticipatory coarticulation does not have a particular structure in joint space: Differences in the joint vector trajectory observed when the following movement segment differs lie equally in directions in joint space that affect and that do not affect the hand’s trajectory in space. This observation is consistent with the idea that motor planning takes place in terms of the spatial goals of a movement. That idea comes from neurophysiological evidence that neural populations in motor and premotor cortex code for the direction of the hand’s movement in space (Georgopoulos et al 1986). This is consistent with a wealth of behavioral studies that include the apparent regularity and invariance of end-effector kinematics (Morasso 1981; Viviani and Flash 1995), as well as with the ideomotor principle according to which movements are planned in the same coordinates in which they are perceived (for review, see Thomaschke 2012).

If anticipatory coarticulation originates in differences in movement planning and movement planning takes place in terms of the hand’s trajectory in space, why do we not observe an “inverse” UCM effect in which more of the joint difference vector lies in the subspace orthogonal to the UCM? The notion of the UCM is that there is no particular process at any level, planning and control, that keeps the UCM components of the joint trajectories invariant or stable (Martin et al 2009). This leads to the UCM structure of variance, but also to motor equivalence and self-motion. An inverse UCM effect for the joint difference vector would, in contrast, presuppose a particular process that would limit covariation in joint space to preserve the UCM component. So differences in planning that induce differences in the spatial trajectories of the hand will show up in the subspace orthogonal to the UCM, but will generically also spread to the UCM subspace itself.

The interpretation of anticipatory coarticulation as reflective of motor planning is consistent with its time course: increasing in time toward the moment the subsequent movement segment is initiated. The structure of the time course of anticipatory coarticulation observed in experiment 2 and 3 confirms this interpretation. In experiment 2, the possibility to visually update the movement on approach to the first target location led to a drop in the non-motor equivalent component of the joint difference vector. Removing the possibility for visual feedback in experiment 3 removed that drop. Note, that the motor equivalent component of the joint difference vector was unaffected by this change, reflecting that the visual updating of movement generation does not couple strongly into the UCM subspace.

The opposite temporal signature was observed for carry-over coarticulation which decreases with increasing distance from the preceding movement segment. Carry-over coarticulation may reflect differences in both planning and control. Our cursory analysis of carry-over coarticulation at the level of individual joint trajectories (Fig. 6) hinted at motor control as a cause of carry-over coarticulation: differences in joint configuration that are induced by the differing first sub-movements tend to persist during the second sub-movement. This was borne out by the UCM analysis of carry-over coarticulation that showed clear signatures of motor equivalence, substantially more of the difference joint vector lies in the UCM subspace than orthogonal to it. That outcome of experiment 1 was a replication of our earlier findings (Hansen et al 2015). It is consistent with the UCM principle of movement generation, in which control processes counteract deviations at the end-effector level, but less so at the level of the motor equivalent joint configurations (Martin et al 2009). This outcome is also consistent with a literature on hysteresis effects in pointing movements (e.g., Cruse et al 1993).

While the observed UCM structure of carry-over coarticulation is consistent with principles of motor control, it does not preclude that movement planning may contribute to carry-over coarticulation. The component of the joint difference vector orthogonal to the UCM (carry-over coarticulation at the end-effector level) is not zero and may reflect such a planning component. In fact, van der Wel et al (2007) observed both anticipatory and carry-over coarticulation at the level of the hand’s trajectory in space in a task in which the hand performed a sequential movement task that involved obstacle avoidance. These authors did not examine these coarticulation effects in joint space, so they could not discover the different joint space signatures of carry-over vs. anticipatory coarticulation reported here.

### Comparison of limb with speech articulatory movements

Speech articulatory gestures are redundant in the sense that multiple configurations of the articulatory apparatus may give rise to the same sound (Guenther 2016). The acoustic signal may be considered the task or performance variable. So a notion of motor-equivalence could be invoked for speech production analogously as done here (Schöner et al 2008). Coarticulation effects in speech production have been reported to affect both articulator configurations and the acoustic signal. If coarticulation in speech production is systematically motor equivalent and if that signature differs between carry-over and anticipatory coarticulation as reported here, is not established at this point. It may be interesting to study these questions using the formal machinery of the UCM method of analysis (Schöner et al 2008).

In speech production, researchers have examines coarticulation effects with respect to the metric similarity between the neighboring phonemes and associated articulatory gestures. Assimilation is most commonly reported, in which neighboring sounds are generated in a more similarly way than if they are produced in a different context (Ohala 1993). In our setting, assimilation may be examined at the level of the performance variable, the objects spatial path. For experiment two and three we examined the object’s spatial position at the end of the first segment in anticipatory coarticulation. We found that about 70% of participants put the object down on the first target in a position that was shifted way from the direction to the next target, a form of dissimilation. About 10% of participants put the object down closer to the next target, a form of assimilation. In about 20% of participants, not systematic effect was observable at the object level. All of these effects were very small, however ($<\,2$ mm for exp. 2, $<\,5$ mm for exp. 3). So the analogy between speech and limb movements has limits (Grimme et al 2011). The main insight we obtained here is that even in naturalistic and relatively slow limb movements, more than one movement segment are prepared at a time and neighboring movement segments influence each other.

## Conclusion

We have observed anticipatory coarticulation in a two-segment naturalistic movement task. The differences in joint vector trajectories observed when the upcoming movement differs are equally distributed in directions of joint space in which the hand’s position in space is invariant and directions in joint space in which it is not. We argued that this was consistent with anticipatory coarticulation reflecting processes of movement planning that reside at the level of the hand’s trajectory in space.

We have replicated carry-over coarticulation in the same experiment, confirming that the differences in joint vector trajectories observed when the preceding movement differs lie most strongly in directions of joint space in which the hand’s position in space is invariant. This UCM signature of carry-over coarticulation is consistent with the hypothesis that carry-over coarticulation reflects the properties of processes of motor control.
